# Autoimmune Disease is Increased in Women With Primary Ovarian Insufficiency

**DOI:** 10.1210/clinem/dgae828

**Published:** 2024-11-28

**Authors:** Victoria Wang, Jessica A Walsh, JoAnn Zell, Lauren E Verrilli, Joseph M Letourneau, Erica B Johnstone, Kristina Allen-Brady, Corrine K Welt

**Affiliations:** Division of Endocrinology, Metabolism and Diabetes, Department of Internal Medicine, University of Utah School of Medicine, Salt Lake City, UT 84112, USA; Division of Rheumatology, Department of Internal Medicine, University of Utah School of Medicine, Salt Lake City, UT 84112, USA; Division of Rheumatology, Department of Internal Medicine, University of Colorado, Aurora, CO 80045, USA; Division of Reproductive Endocrinology and Infertility, Department of Obstetrics and Gynecology, University of Utah School of Medicine, Salt Lake City, UT 84112, USA; Intermountain Healthcare, Murray, UT 84107, USA; Division of Reproductive Endocrinology and Infertility, Department of Obstetrics and Gynecology, University of Utah School of Medicine, Salt Lake City, UT 84112, USA; Division of Reproductive Endocrinology and Infertility, Department of Obstetrics and Gynecology, University of Utah School of Medicine, Salt Lake City, UT 84112, USA; Division of Epidemiology, Department of Internal Medicine, University of Utah School of Medicine, Salt Lake City, UT 84108, USA; Division of Endocrinology, Metabolism and Diabetes, Department of Internal Medicine, University of Utah School of Medicine, Salt Lake City, UT 84112, USA

**Keywords:** systemic lupus erythematosus, psoriasis, thyroid, menopause

## Abstract

**Context:**

Autoimmune disease is common in women with primary ovarian insufficiency (POI), and the genetic etiology of autoimmune disease suggests that it could be hereditary in families of women with POI.

**Objective:**

We hypothesized that a subset of women with POI and their family members would have an increased risk for autoimmune disorders.

**Design:**

Population-based study using electronic health records from 1995 to 2022.

**Setting:**

Two major Utah healthcare systems serving 85% of the state.

**Subjects:**

Women with POI (n = 610) were identified using International Classification of Diseases (ICD) codes and charts were reviewed for accuracy. First-, second-, and third-degree relatives were identified using genealogy data in the Utah Population Database.

**Intervention:**

Autoimmune diagnoses were identified using ICD codes.

**Main Outcome Measures:**

The relative risk of autoimmune disease in women with POI and relatives was estimated by comparison to population rates.

**Results:**

At least 1 autoimmune disease was identified in 25% of women with POI. The relative risk of autoimmune hypothyroidism (odds ratio [95% confidence interval] 6.88 [5.71, 8.22]; *P* < .001), adrenal insufficiency (4.72 [1.73, 10.28]; *P* = .0020), type 1 diabetes (4.13 [2.14, 7.22]; *P* = 5.25X10^−5^), rheumatoid arthritis (5.66 [3.10, 9.50]; *P* = 3.70X10^−7^), vitiligo (15.33 [6.16, 31.58]; *P* = 5.25X10^−7^), celiac disease (7.58 [3.47, 14.39]; *P* = 4.47X10^−6^), psoriasis (3.90 [2.01, 6.81]; *P* = 9.04X10^−5^) and systemic lupus erythematosus (4.43 [1.63, 9.64]; *P* = .0027) were increased in women with POI compared to population rates. There was no increased risk of autoimmune disease in family members.

**Conclusion:**

Data confirm increased autoimmune disease in women with POI. The increased risk is largely related to autoimmune polyglandular syndrome types 1 through 4 and autoimmune hypothyroidism. The absence of risk in family members may result from differences in environmental influences or hormone milieu.

Primary ovarian insufficiency (POI) is diagnosed when a woman, less than 40 years old, has amenorrhea for at least 4 months, elevated FSH (in the menopausal range), and low estradiol levels (1). The etiology of POI includes genetic, autoimmune, environmental, and iatrogenic causes. Autoimmune disease typically manifests during reproductive age, and ovarian autoimmunity has been estimated to play a role in 4.5% to 5% of POI cases when defined using adrenal cortical antibody measurements (2-4). When all autoimmune disease is considered in association with POI, the rates are as high as 15% to 20%, largely driven by thyroid autoimmune disease (5-7).

Women with POI have a higher frequency of autoimmune disorders than women in the general population (7). Adrenal autoimmunity and adrenal insufficiency have the strongest relationship and can occur in the context of autoimmune polyglandular syndromes (APS). When POI is associated with adrenal autoimmunity or insufficiency, true autoimmune oophoritis can occur (7, 8). In fact, histologically proven ovarian autoimmunity or oophoritis has only been documented in the presence of autoimmune adrenal insufficiency or in the presence of adrenal autoantibodies (7, 8).

Nonadrenal autoimmunity has also been associated with POI (7). However, the well-described ovarian histologic alterations seen in adrenal-driven disease have not been clearly demonstrated, suggesting that systemic autoimmune disease could cause POI via other mechanisms related to lack of self-tolerance. The associated autoimmune diseases target other specific endocrine organs [hypothyroidism, hypoparathyroidism, type 1 diabetes mellitus (T1DM)], nonendocrine organs such as synovial tissue [rheumatoid arthritis (RA)] or can be systemic nonendocrine disease targeting a variety of tissues [systemic lupus erythematosus (SLE), Sjogren's syndrome] (7, 9). A cause for the relationship between these nonovarian autoimmune disorders and POI remains unclear.

The heritability of POI has been demonstrated through a high prevalence of familial cases, with the highest risk in first-degree relatives (10). Similarly, autoimmune disease frequently appears to have a heritable contribution with a 14% to 40% risk of sharing SLE in identical twins as an example (11, 12). The polygenic risk for autoimmune disease explains this heritability pattern, as there are over 150 identified gene loci that may lead to the development of lupus (13). Patients with 1 autoimmune syndrome often exhibit features of other autoimmune diseases perhaps due to genetic predisposition (14). However, even in families with close relatives who carry autoimmune disease unrelated to POI, the risk of autoimmune disease is only 2% (13, 15). Therefore, it is of interest to determine whether POI and autoimmune disease exhibit shared familial clustering.

We hypothesized that autoimmune diagnoses would be found at a greater frequency in women with POI. We chose endocrine and other autoimmune disorders that form the components of APS and non-APS disorders that have a more variable relationship with POI depending on the study design (5-7). Based on the heritability of POI and autoimmune diseases, we also hypothesized an increased risk of autoimmune diseases in first-degree, second-degree, and third-degree relatives.

## Materials and Methods

### Subjects

We identified women with POI using electronic medical records at the University of Utah Health and Intermountain Healthcare from 1995 to 2022. The cases of POI were obtained through International Classification of Disease (ICD) codes: ICD-9 (256.3, 256.31, 256.39) and ICD-10 codes (E28.3, E28.31, E28.39, E28.310, and E28.319) and electronic medical record notes indicating POI diagnoses and/or lab values (elevated FSH > 20 IU/L or anti-Müllerian hormone <0.08 ng/mL in a woman under the age of 40 years at the time of the laboratory draw). Subjects with the following current procedural terminology codes were excluded: hysterectomy, oophorectomy, endometriosis with pelvic surgery, pelvic radiation or chemotherapy before the diagnosis of POI, and Turner syndrome (ICD-9 758.6 and ICD-10 Q96).

Those identified with POI ICD codes were verified through individual chart review by a medical or reproductive endocrinologist (C.K.W. or L.E.V.) for inclusion. Validation factors included lab values (FSH and anti-Müllerian hormone levels), diagnosis details, POI signs, and the type of physician making the diagnosis (10).

### Pedigree Creation

Medical record numbers for women with POI were converted to Utah Population Database (UPDB) identification numbers by an independent oversight group under the requirements for use. Subsequently, UPDB IDs were linked to genealogy within the UPDB. For the familial clustering analysis, POI probands were required to have at least 3 generations of genealogy information available (proband, both parents, and 3 out of 4 grandparents). Three or more generations increases the likelihood that subjects and their families have lived in Utah for many generations and would have complete family data and health care data.

### Ethics

The institutional review boards at the University of Utah and Intermountain Healthcare approved this study.

### Identification of Autoimmune Diseases

We identified autoimmune diseases using ICD codes in women with POI and their relatives, choosing those found in previous studies and known polyglandular syndromes (7, 16). These included adrenal deficiencies (ICD-9 255.X, ICD-10 27.X), hypoparathyroidism (252.1, E20.X), type 1 diabetes mellitus (250.X, E10.X), vitiligo (709.01, L80), celiac disease (579.0, K90.0), vitamin B12 deficiencies (281.0, D51.X), multiple sclerosis (340, G35), systemic lupus erythematosus (710.0, M32.X), Crohn's disease (555.X, K50.X), psoriasis (696.X, L40.X), ulcerative colitis (556.X, K51.X), inflammatory bowel disease (569.89), systemic sclerosis (701.0, M34.X), scleroderma (701.0, M34.X), RA (714.0, M05.X), hypothyroidism (244.8, 244.9, E03.X, E06.X), immune thrombocytopenia (287.31, D69.3), Behcet (136.1, M35.2), myasthenia gravis (358.01, G70.X), and primary biliary cirrhosis (571.6, K74.3). Autoimmune disease in women with POI was verified through individual chart review and discussed with clinical rheumatologists (J.W. and J.Z.).

### Relative Risk Analysis

The relative risk of individual autoimmune diseases in women with POI and their relatives was analyzed. First-degree relatives included parents, siblings, and children. Second-degree relatives included grandparents, aunts/uncles, nieces/nephews, half-siblings, and grandchildren. Third-degree relatives included great-grandparents, great-grandchildren, and first cousins.

The relative risk of a disease estimates the likelihood an individual may develop a disease if the woman has POI or is a relative of a woman with POI. The relative risk was calculated as a ratio of the observed number of a specific type of autoimmune disease for a woman with POI or a specific relative type compared to the expected number of autoimmune disease cases for a woman or a specific relative type. To obtain the expected number of autoimmune disease cases, population rates were calculated for each 5-year birth cohort represented by the POI subjects or relatives and birthplace (Utah vs outside of Utah) within the University of Utah Health and Intermountain Healthcare. The rate was defined to be the total number of cases of a specific autoimmune disease divided by the total cohort size. The number of expected cases was then calculated by summing each cohort-specific autoimmune disease risk for each individual in a set of relatives of a specific type (eg, first-degree relatives). Approximate 95% confidence intervals and exact hypothesis tests of the null hypothesis (relative risk = 1.0) were constructed assuming that the number of autoimmune disease cases found among the relatives follows a Poisson distribution. The studies performed with the UPDB are population based, which reduces the risk for sampling bias that can occur due to proband identification and oversampling from pedigrees with multiple affected members. We corrected for multiple testing for 18 autoimmune disorders (*P* < .05/18 = 0.0028).

## Results

After a thorough search of diagnostic codes and chart review at the University of Utah and Intermountain Healthcare, 610 women were identified with a diagnosis of POI. The average age (± SD) at the time of POI diagnosis was 32.7 ± 7.4 years, and the average age at the time of the study was 48.3 ± 11.9 years (range 14-93 years). Race/ethnicity of the women were self-reported. The distribution was predominantly White (78.7%), White Hispanic (10.5%), Asian (1.6%), and multiple races (3.8%). The remaining encompassed Native American, Pacific Islander, Black, and unknown. Of the 610 women, 410 had 3 generations of genealogical data available in the UPDB. These 410 women had 2271 first-degree relatives, 5683 second-degree relatives, and 11 697 third-degree relatives.

Of the 610 women, we identified 153 subjects with any autoimmune disease (25%). Women with POI had an increased risk for several autoimmune diseases compared to population rates of autoimmune diseases (Table 1). Of the autoimmune diseases identified, most of the women had hypothyroidism (79% of the women with autoimmune disease). Fewer than 10% of those with autoimmune disease had each of the following: RA, vitiligo, celiac disease, T1DM, psoriasis, adrenal insufficiency, and SLE. Of the total with autoimmune disease, 117 women (78%) had 1 autoimmune disease and 36 women (22%) had multiple autoimmune diagnoses. Among the subjects with multiple autoimmune diseases, there were ≤10 women with adrenal insufficiency. All women with adrenal insufficiency met the criteria for autoimmune polyglandular syndrome type 1 if there was an associated diagnosis of hypoparathyroidism or autoimmune polyglandular syndrome type 2 with associated disorders including hypothyroidism, vitiligo, T1DM, and celiac disease. The remaining subjects had various combinations of autoimmune diseases, including APS types 3 and 4, and other combinations that did not fit into APS classifications (Table 2). The age at diagnosis for most of the autoimmune diseases was as expected based on population ages and the literature (Supplementary Table S1) (17).

**Table 1.** Autoimmune disease identified in women with POI (n = 610)

Autoimmune disease	Observed #	Expected #	Relative risk (95% CI)	*P*-value
Hypothyroidism	121	17.60	6.88 (5.71, 8.22)	<.001*
Rheumatoid arthritis	14	2.47	5.66 (3.10, 9.50)	3.70E-07*
Vitiligo	≤ 10	0.46	15.33 (6.16, 31.58)	5.52E-07*
Celiac disease	≤ 10	1.19	7.58 (3.47, 14.39)	4.47E-06*
Type 1 diabetes	12	2.90	4.13 (2.14, 7.22)	5.25E-05*
Psoriasis	12	3.08	3.90 (2.01, 6.81)	9.04E-05*
Adrenal deficiencies	≤ 10	1.27	4.72 (1.73, 10.28)	.0020*
Systemic lupus Erythematosus	≤ 10	1.35	4.43 (1.63, 9.64)	.0027*
Myasthenia gravis	≤ 10	0.09	22.97 (2.78, 82.98)	.0036
Pernicious anemia	≤ 10	0.47	6.45 (1.33, 18.84)	.012
Scleroderma	≤ 10	0.89	3.39 (0.70, 9.90)	.060
Primary biliary cirrhosis	≤ 10	0.12	8.59 (0.22, 47.84)	.11
Hypoparathyroidism	≤ 10	0.17	5.76 (0.15, 32.11)	.16
immune thrombocytopenia	≤ 10	0.24	4.13 (0.11, 23.04)	.21
Multiple sclerosis	≤ 10	2.28	1.76 (0.48, 4.50)	.30
Ulcerative colitis	≤ 10	1.05	1.91 (0.23, 6.90)	.63

The observed number of cases with autoimmune disease, expected number based on population rates, and the relative risk. Based on the Utah Population Database regulations for oversight by the Utah Resource for Genetic and Epidemiologic Research, observed counts < 10 were not listed to protect the identity of the patients. No women with POI were found to have Crohn’s disease, systemic sclerosis, or Behcet disease.

Abbreviations: CI, confidence interval; POI, primary ovarian insufficiency.

Bonferroni multiple testing correction. *P-value < .0028.

**Table 2.** Observed Number of Women with POI and more Than 1 Autoimmune Diseases (n = 36)

Autoimmune diseases	Observed #	Percent
APS type 1 or 2	≤ 10	≤ 1.6
Hypothyroidism ± celiac, myasthenia gravis, MS, SLE, RA, psoriasis, ulcerative colitis, and/or scleroderma or APS type 4	18	3.0
Hypothyroidism and type 1 diabetes ± RA or vitiligo or APS type 3	≤ 10	≤ 1.6
Type 1 diabetes ± RA and/or celiac	≤ 10	≤ 1.6
RA± ulcerative colitis, primary biliary sclerosis, and/or scleroderma	≤ 10	≤ 1.6
SLE ± pernicious anemia	≤ 10	≤ 1.6

Abbreviations: APS, autoimmune polyglandular syndrome; MS, multiple sclerosis; POI, primary ovarian insufficiency; RA, rheumatoid arthritis; SLE, systemic lupus erythematosus

We looked for confirmatory antibody testing for autoimmune disease by chart review. Of the total number of women with POI, only 42 had 21 hydroxylase antibody testing or adrenal insufficiency. In those women with POI and antibody testing who did not have a known diagnosis of adrenal insufficiency before or at the same time as the diagnosis of POI, only 2 women had positive 21 hydroxylase antibodies, but they were only minimally positive. One subject was negative on repeat testing, and the other had a different known genetic diagnosis. For other autoimmune diagnoses, 23 women with hypothyroidism had antibody testing and 9 (39%) were found to be positive; 8 women with RA had antibody testing and 4 (39%) were positive; 2 women, each, with T1DM and celiac disease had antibodies tested and 1 (50%) was positive for each; 4 women with SLE had antibody testing and 2 (50%) were positive; and for myasthenia gravis, 1 woman was tested with 100% positive.

We separately investigated the risk of the autoimmune conditions in the 410 women with POI and 3 or more generations of genealogical data. We observed similar increased risks of the same autoimmune diseases with POI as in the complete POI cohort. Two exceptions were SLE, which was not significant after correcting for multiple testing, and myasthenia gravis, which was found to be associated with POI (Table 3). There was no increased risk of autoimmune disease in the relatives of women with POI after correction for multiple testing (Table 4).

**Table 3.** Autoimmune disease identified in the subset of women with POI with 3 or more generations of genealogical data (n = 410)

Autoimmune disease	Observed #	Expected #	Relative risk (95% CI)	*P*-value
Hypothyroidism	83	12.05	6.89 (5.49, 8.54)	<.001*
Celiac disease	≤ 10	0.92	8.71 (3.76, 17.16)	5.59E-06*
Rheumatoid arthritis	≤ 10	1.61	6.21 (2.98, 11.42)	7.57E-06*
Psoriasis	11	2.01	5.46 (2.73, 9.77)	8.89E-06*
Type 1 diabetes	≤ 10	2.10	4.77 (2.29, 8.77)	6.82E-05*
Vitiligo	≤ 10	0.28	14.51 (3.95, 37.15)	1.93E-04*
Adrenal deficiencies	≤ 10	0.85	5.90 (1.91, 13.76)	.0018*
Myasthenia gravis	≤ 10	0.06	30.83 (3.73, 111.38)	.0020*
Systemic lupus Erythematous	≤ 10	0.93	4.31 (1.18, 11.04)	.015
Anemia and B12 deficiencies	≤ 10	0.29	6.94 (1.23, 25.07)	.034
Hypoparathyroidism	≤ 10	0.11	9.17 (0.23, 51.09)	.10
immune thrombocytopenia	≤ 10	0.18	5.64 (0.14, 31.43)	.16
Ulcerative colitis	≤ 10	0.74	2.69 (0.33, 9.72)	.17
Multiple sclerosis	≤ 10	1.60	1.87 (0.39, 5.47)	.42
Scleroderma	≤ 10	0.62	1.60 (0.04, 8.92)	1.00

The observed number of cases with autoimmune disease, expected number based on population rates, and the relative risk. Based on regulations of the Utah Population Database oversight by the Utah Resource for Genetic and Epidemiologic Research, observed numbers were listed as < 10 to protect the identity of the patients. No women were found to have Crohn's disease, systemic sclerosis, Behcet's disease, or primary biliary cirrhosis.

Abbreviations: CI, confidence interval; POI, primary ovarian insufficiency.

Bonferroni multiple testing correction. *P-value < .0028.

**Table 4.** Relative risk of autoimmune disease among first-degree, second-degree, and third-degree relatives of women with POI

Relative type	Autoimmune disease	Observed #	Expected #	Relative risk (95% CI)	*P*-value
First degree (n = 2271)	Hypothyroidism	68	48.78	1.39 (1.08, 1.77)	.0069
	Celiac disease	≤ 10	3.55	1.12 (0.31, 2.88)	1.00
	Rheumatoid arthritis	≤ 10	8.69	1.15 (0.55, 2.12)	.73
	Psoriasis	11	9.83	1.12 (0.56, 2.00)	.75
	Type 1 diabetes	15	13.20	1.14 (0.64, 1.87)	.68
	Vitiligo	≤ 10	1.46	2.74 (0.75, 7.03)	.061
	Adrenal deficiencies	≤ 10	5.29	1.51 (0.65, 2.98)	.27
	Crohn’s disease	≤ 10	3.56	1.40 (0.46, 3.28)	.60
	Systemic lupus Erythematous	≤ 10	3.02	0.33 (0.01, 1.85)	.28
	Anemia and B12 deficiencies	≤ 10	1.57	1.27 (0.15, 4.60)	1.00
	Hypoparathyroidism	≤ 10	0.65	6.16 (1.68, 15.78)	.0044
	Behcet	≤ 10	0.17	5.88 (0.15, 32.78)	.16
	Ulcerative colitis	≤ 10	4.58	1.09 (0.35, 2.55)	1.00
	Multiple sclerosis	≤ 10	5.97	1.51 (0.69, 2.86)	.21
	Scleroderma	≤ 10	2.47	1.62 (0.44, 4.15)	.32
Second degree (n = 5683)	Hypothyroidism	146	124.4	1.17 (0.99, 1.38)	.054
	Celiac disease	≤ 10	7.70	1.30 (0.62, 2.39)	.47
	Rheumatoid Arthritis	32	23.94	1.34 (0.91, 1.89)	.10
	Psoriasis	23	20.15	1.14 (0.72, 1.71)	.58
	Type 1 diabetes	43	31.65	1.36 (1.04, 1.83)	.050
	Vitiligo	≤ 10	3.14	1.27 (0.35, 3.26)	.78
	Adrenal deficiencies	18	12.81	1.41 (0.83, 2.22)	.16
	Crohn’s disease	11	6.80	1.61 (0.81, 2.89)	.12
	Systemic lupus Erythematous	≤ 10	6.27	1.28 (0.55, 2.51)	.54
	Anemia and B12 deficiencies	≤ 10	4.93	0.41 (0.049, 1.47)	.26
	Hypoparathyroidism	≤ 10	1.63	1.84 (0.38, 5.37)	.42
	Systemic sclerosis	≤ 10	1.15	1.74 (0.21, 6.30)	.64
	Ulcerative colitis	18	10.39	1.73 (1.03, 2.74)	.022
	Multiple sclerosis	13	11.38	1.14 (0.61, 1.95)	.65
	Scleroderma	≤ 10	6.13	1.47 (0.67, 2.79)	.31
	Immune thrombocytopenia	≤ 10	2.09	0.96 (0.12, 3.46)	1.00
	Primary biliary cirrhosis	≤ 10	1.34	1.49 (0.18, 5.39)	.65
Third degree (n = 11 697)	Hypothyroidism	267	235.00	1.14 (1.00, 1.28)	.037
	Celiac disease	18	14.89	1.21 (0.72, 1.91)	.43
	Rheumatoid arthritis	52	41.70	1.25 (0.93, 1.64)	.12
	Psoriasis	36	41.32	0.87 (0.61, 1.21)	.44
	Type 1 diabetes	67	61.40	1.09 (0.85, 1.39)	.48
	Vitiligo	≤ 10	5.81	0.17 (0.0044, 0.96)	.056
	Adrenal deficiencies	27	21.42	1.26 (0.83, 1.83)	.23
	Crohn’s disease	14	14.60	0.96 (0.52, 1.61)	.90
	Systemic lupus Erythematous	≤ 10	11.87	0.59 (0.24, 1.22)	.19
	Anemia and B12 deficiencies	≤ 10	9.02	0.55 (0.18, 1.29)	.19
	Hypoparathyroidism	≤ 10	2.90	1.38 (0.36, 3.53)	.54
	Ulcerative colitis	21	19.41	1.08 (0.67, 1.65)	.73
	Multiple sclerosis	22	20.93	1.05 (0.66, 1.59)	.83
	Scleroderma	≤ 10	10.96	0.91 (0.44, 1.68)	.88
	Systemic sclerosis	≤ 10	1.96	0.51 (0.013, 2.84)	.73
	Immune thrombocytopenia	≤ 10	3.50	1.14 (0.31, 2.93)	1.00
	Myasthenia gravis	≤ 10	1.78	0.56 (0.014, 3.13)	.73
	Primary biliary cirrhosis	≤ 10	2.13	0.47 (0.012, 2.62)	.54

There was no significant increase in the relative risk of any autoimmune disease. For relative groups missing any of the autoimmune diseases, there were no cases identified.

Abbreviations: CI, confidence interval; POI, primary ovarian insufficiency.

Bonferroni multiple testing correction. *P-value < .0028.

## Discussion

Our data demonstrate autoimmune disease in 25% of women with POI and an increased relative risk for multiple autoimmune disorders in women with POI, confirming previous findings (18). Women with multiple autoimmune disorders presented with components of autoimmune polyglandular syndrome types 1 through 4. Our data do not demonstrate an increased relative risk for the same autoimmune disorders in family members of women with POI. These data suggest that other factors in addition to shared POI and autoimmune genetic risk play a role in the increased autoimmune disease burden in POI.

### Endocrine Autoimmunity

Our data demonstrate an increase in adrenal insufficiency in the context of APS types 1 and 2, as expected based on the known relationship between adrenal and ovarian autoimmunity (19). APS type 1 is characterized by a triad of Addison's disease, chronic mucocutaneous candidiasis, and hypoparathyroidism, due to mutations in the autoimmune regulatory gene, *AIRE* (16). APS type 2 is a cluster of 2 or more endocrine diseases, commonly thyroid disease and T1DM, with a more complex inheritance pattern (16). The prevalence of clinical adrenal insufficiency, ≤ 1.6% in our dataset of women with POI, is similar to that reported in previous studies demonstrating 0.2% of women with POI and adrenal insufficiency (6, 18). In addition, approximately 4.5% to 5% of women with POI will have adrenal autoimmunity without current adrenal insufficiency (3, 20). Many of our subjects did not have available testing for adrenal cortical or 21-hydroxylase antibodies. Therefore, our number with adrenal autoimmunity is likely an underestimate at 2.9%. In patients with autoimmune adrenal failure, antibodies to the adrenal cortex and to steroid cells have been identified by indirect immunofluorescence on fixed tissue samples and best discriminate autoimmune POI from POI of other etiologies (7, 19). Autoimmune adrenal insufficiency or the presence of adrenal autoantibodies are the only autoimmune diseases or antibody manifestations that have been documented in association with histologically proven ovarian autoimmune oophoritis that initially targets the steroid-producing theca cells (7, 8). These infiltrates eventually spill over, destroying the surrounding tissue and resulting in ovarian insufficiency (8).

As demonstrated previously, the most common autoimmune disease was hypothyroidism, with a prevalence of 19.8%, accounting for a component of autoimmune disease in 79% of the women with POI and autoimmune disease in our cohort. Hypothyroidism has been found in 12% to 15% of patients with POI in previous studies (7, 18, 21), slightly lower than in the current data. Thyroid autoimmunity has been referred to as the presence of antibodies to thyroid peroxidase, TSH receptor, and thyroglobulin (22). Previous studies did not report an association between thyroid autoimmunity and fecundity or ovarian reserve (23, 24), suggesting that the relationship is not causal for POI despite the association.

The prevalence of T1DM was 2.0% among women with POI in our study, similar to previous findings (7). The majority of the subjects with T1DM also had hypothyroidism and POI, which has been categorized as APS type 3 (16). Women with T1DM have been found to have lower anti-Mullerian hormone levels, suggesting decreased ovarian reserve (25). The mechanism of ovarian insufficiency could be related to microvascular disease or glycosylation rather than an autoimmune mechanism itself (26). Concomitant hypothyroidism does not appear to be an important causal factor for POI, as discussed earlier.

### Nonendocrine and Systemic Disease

In addition to endocrine autoimmune disease, we found an increased risk for nonendocrine autoimmune diseases including RA, SLE, psoriasis, vitiligo, myasthenia gravis, and celiac disease. In some cases, these nonendocrine autoimmune diseases were found as components of APS types 2 or 3 or APS type 4 in combination with thyroid disease (16).

There has been an established relationship between POI and both RA and SLE. Our study demonstrated a prevalence of 2.3% for RA and ≤1.6% SLE in women with POI. Of note, cyclophosphamide therapy for severe SLE can cause POI with high and cumulative doses (27, 28). However, we removed patients with cyclophosphamide treatment from our cohort after our extensive chart review. Anti enolase and anti corpus luteum antibodies have been identified as ovarian targets in RA and SLE; however, these antibodies have not been reproducibly associated with POI over time and there is no clear relationship between these autoantibodies and an autoimmune infiltrate in the ovary (7, 29, 30). Ovarian vasculitis has also been described, with pathologic evidence described in SLE, and could play a role in ovarian damage (31). Finally, Mendelian randomization suggests a causal genetic relationship between SLE and POI, but replication is needed as the power of the study was likely inadequate (32). Overall, the relationship between RA and SLE and POI remains undetermined.

Similar to previous findings, psoriasis is also increased in women with POI in the current study (2). Psoriasis has been documented as a component of APS types 2 to 4 (16) but has not been reported as frequently in association with POI. Compared to other autoimmune diseases, the underlying mechanism causing psoriasis was previously thought to be distinct, without evidence for autoantibodies or autoreactive T-cells (33). However, more recent data suggest that T cell self-reactivity is a central component of psoriasis pathogenesis, although there are still no associated autoantibodies (33). The differences in the spectrum of immune mechanisms and clinical presentations in psoriasis compared to other autoimmune disease mechanisms could provide additional insights into the relationship with POI.

We did not find familial autoimmune disease in families of women with POI after correction for multiple testing. Autoimmune disease is highly heritable, as demonstrated by higher concordance between monozygotic (13-40%) compared to dizygotic twins for many autoimmune diseases (11-13). Genome-wide association studies have demonstrated complex genetic risk for autoimmune diseases, suggesting that there should be some aggregation in large families like those we studied (14). Previous family studies have demonstrated co-occurrence of autoimmune disease in family members, with the strongest evidence for T1DM, Crohn's disease, psoriasis, and multiple sclerosis (34). However, the familial cases in other diseases may be few, as there are less than 2% of women with SLE who have relatives with SLE, for example (13, 15). Our results may be related to small numbers, which is a problem with rare autoimmune diseases, particularly because we studied them as a secondary entity in our POI families and were not studying autoimmune disease as our entry into the analysis. We may have missed cases if a diagnosis was not made or was documented in a different health system. Developing autoimmune disease also depends on environmental exposures (35). Differences in environmental exposures in women with POI compared to relatives could be a cause, although the environment may be shared especially for first-degree relatives. It is also possible that differences in sex steroid levels, particularly in estrogens and androgens, play a role in the differing disease prevalence in women with POI compared to their family members (15). In particular, changes in estradiol levels can exacerbate SLE, while androgens suppress autoimmunity in experimental models (15, 17).

It is also possible that familial aggregation may be more complicated, with 1 autoimmune disease found in a woman with POI and different autoimmune diseases in family members. Genetic studies demonstrate overlap in genetic loci among several autoimmune diseases, and at least 40% of genetic risk loci influence more than 1 autoimmune disease (14). For example, loci conferring risk for more than 1 autoimmune disease include the *STAT4* locus for RA and SLE; the *CD289-CTLA4* locus for T1DM and celiac disease or RA; the *ICAM3* locus for RA with SLE; the *TYK2* locus for RA with SLE, T1DM, and inflammatory bowel disease; and the *RGS1* locus for T1DM and multiple sclerosis and celiac disease (14). In line with gene loci overlapping for different autoimmune diseases, a familial relationship among 2 or more different autoimmune diseases has been demonstrated in some studies (36). We did not examine these more complicated autoimmune disease relationships in our families.

Strengths of this study include the careful case definition of POI at a population level validated with chart review and the extensive genealogy database. Our results are generalizable to other northern European populations. Although 10.4% of these women had Hispanic ethnicity (37), our results may not be generalizable to other races and ethnicities. Other limitations include our inability to identify all autoimmune conditions in the relatives, although we did use data from the 2 largest health care systems in Utah. We may also miss autoimmune disease based on miscoding and miss antibody testing based on the availability of data in the medical records.

Our data demonstrate an increased relative risk for several autoimmune diseases in women with POI compared to the general population. The absence of increased risk in family members does not mean that there is no genetic predisposition to autoimmune disease in POI, but it suggests epigenetic, environmental, and/or hormonal risk may increase the likelihood of autoimmune development in women with POI. These findings support clinical practice to expand screening for autoimmune diseases in women with POI as already recommended for hormones and antibodies associated with adrenal and thyroid disease.

## Data Availability

The data supporting the current study have not been deposited in a public repository because they require institutional review board approval and Utah Resource for Genetic and Epidemiology Research approval but are available from the corresponding author on request.

## References

[1] Welt CK . Primary ovarian insufficiency: a more accurate term for premature ovarian failure. Clin Endocrinol. 2008;68(4):499‐509. 10.1111/j.1365-2265.2007.03073.x 17970776

[2] Jiao X, Zhang H, Ke H, et al Premature ovarian insufficiency: phenotypic characterization within different etiologies. J Clin Endocrinol Metab. 2017;102(7):2281‐2290. 28368522 10.1210/jc.2016-3960

[3] Vogt EC, Real FG, Husebye ES, et al Premature menopause and autoimmune primary ovarian insufficiency in two international multi-center cohorts. Endocr Connect. 2022;11(5):e220024. 35521804 10.1530/EC-22-0024 PMC9175594

[4] Rosenblum MD, Remedios KA, Abbas AK. Mechanisms of human autoimmunity. J Clin Invest. 2015;125(6):2228‐2233. 25893595 10.1172/JCI78088 PMC4518692

[5] Jiao X, Meng T, Zhai Y, et al Ovarian reserve markers in premature ovarian insufficiency: within different clinical stages and different etiologies. Front Endocrinol (Lausanne). 2021;12:601752. 33815272 10.3389/fendo.2021.601752 PMC8015703

[6] Ishizuka B . Current understanding of the etiology, symptomatology, and treatment options in premature ovarian insufficiency (POI). Front Endocrinol (Lausanne). 2021;12:626924. 33716979 10.3389/fendo.2021.626924 PMC7949002

[7] Hoek A, Schoemaker J, Drexhage HA. Premature ovarian failure and ovarian autoimmunity. Endocr Rev. 1997;18(1):107‐134. 9034788 10.1210/edrv.18.1.0291

[8] Bakalov VK, Anasti JN, Calis KA, et al Autoimmune oophoritis as a mechanism of follicular dysfunction in women with 46,XX spontaneous premature ovarian failure. Fertil Steril. 2005;84(4):958‐965. 16213850 10.1016/j.fertnstert.2005.04.060

[9] Silva CA, Yamakami LY, Aikawa NE, Araujo DB, Carvalho JF, Bonfa E. Autoimmune primary ovarian insufficiency. Autoimmun Rev. 2014;13(4-5):427‐430. 24418305 10.1016/j.autrev.2014.01.003

[10] Verrilli L, Johnstone E, Welt CK, Allen-Brady K. Primary ovarian insufficiency has strong familiality: results of a multigenerational genealogical study. Fertil Steril. 2023;119(1):128‐134. 36283864 10.1016/j.fertnstert.2022.09.027 PMC10024920

[11] Jarvinen P, Aho K. Twin studies in rheumatic diseases. Semin Arthritis Rheum. 1994;24(1):19‐28. 7985034 10.1016/0049-0172(94)90096-5

[12] Surace AEA, Hedrich CM. The Role of Epigenetics in autoimmune/inflammatory disease. Front Immunol. 2019;10:1525. 31333659 10.3389/fimmu.2019.01525 PMC6620790

[13] Harroud A, Hafler DA. Common genetic factors among autoimmune diseases. Science. 2023;380(6644):485‐490. 37141355 10.1126/science.adg2992 PMC13069990

[14] Lincoln MR, Connally N, Axisa PP, et al Genetic mapping across autoimmune diseases reveals shared associations and mechanisms. Nat Genet. 2024;56(5):838‐845. 38741015 10.1038/s41588-024-01732-8 PMC13095137

[15] Rubtsov AV, Rubtsova K, Kappler JW, Marrack P. Genetic and hormonal factors in female-biased autoimmunity. Autoimmun Rev. 2010;9(7):494‐498. 20144912 10.1016/j.autrev.2010.02.008 PMC3171140

[16] Frommer L, Kahaly GJ. Autoimmune Polyendocrinopathy. J Clin Endocrinol Metab. 2019;104(10):4769‐4782. 31127843 10.1210/jc.2019-00602

[17] Welt CK . Autoimmune disease is increased in women with primary ovarian insufficiency Supplementary Table 1. 2024. Figshare. Figure. 10.6084/m9.figshare.27531054.v2 PMC12261096 39607709

[18] Bakalov VK, Gutin L, Cheng CM, et al Autoimmune disorders in women with turner syndrome and women with karyotypically normal primary ovarian insufficiency. J Autoimmun. 2012;38(4):315‐321. 22342295 10.1016/j.jaut.2012.01.015 PMC3358475

[19] Falorni A, Laureti S, Candeloro P, et al Steroid-cell autoantibodies are preferentially expressed in women with premature ovarian failure who have adrenal autoimmunity. Fertil Steril. 2002;78(2):270‐279. 12137862 10.1016/s0015-0282(02)03205-3

[20] La Marca A, Brozzetti A, Sighinolfi G, Marzotti S, Volpe A, Falorni A. Primary ovarian insufficiency: autoimmune causes. Curr Op Ob Gyn. 2010;22(4):277‐282. 10.1097/GCO.0b013e32833b6c70 20543691

[21] Goswami R, Marwaha RK, Goswami D, et al Prevalence of thyroid autoimmunity in sporadic idiopathic hypoparathyroidism in comparison to type 1 diabetes and premature ovarian failure. J Clin Endocrinol Metab. 2006;91(11):4256‐4259. 16895958 10.1210/jc.2006-1005

[22] Frohlich E, Wahl R. Thyroid autoimmunity: role of anti-thyroid antibodies in thyroid and extra-thyroidal diseases. Front Immunol. 2017;8:521. 28536577 10.3389/fimmu.2017.00521 PMC5422478

[23] Plowden TC, Schisterman EF, Sjaarda LA, et al Subclinical hypothyroidism and thyroid autoimmunity are not associated with fecundity, pregnancy loss, or live birth. J Clin Endocrinol Metab. 2016;101(6):2358‐2365. 27023447 10.1210/jc.2016-1049 PMC4891792

[24] Osuka S, Iwase A, Goto M, et al Thyroid autoantibodies do not impair the ovarian reserve in euthyroid infertile women: a cross-sectional study. Horm Metab Res. 2018;50(7):537‐542. 29991084 10.1055/a-0637-9430

[25] Soto N, Iniguez G, Lopez P, et al Anti-Mullerian hormone and inhibin B levels as markers of premature ovarian aging and transition to menopause in type 1 diabetes mellitus. Hum Reprod. 2009;24(11):2838‐2844. 19643804 10.1093/humrep/dep276

[26] Tatone C, Amicarelli F, Carbone MC, et al Cellular and molecular aspects of ovarian follicle ageing. Hum Reprod Update. 2008;14(2):131‐142. 18239135 10.1093/humupd/dmm048

[27] Huong DL, Amoura Z, Duhaut P, et al Risk of ovarian failure and fertility after intravenous cyclophosphamide. A study in 84 patients. J Rheumatol. 2002;29(12):2571‐2576. 12465154

[28] Spears N, Lopes F, Stefansdottir A, et al Ovarian damage from chemotherapy and current approaches to its protection. Hum Reprod Update. 2019;25(6):673‐693. 31600388 10.1093/humupd/dmz027 PMC6847836

[29] Forges T, Monnier-Barbarino P, Faure GC, Bene MC. Autoimmunity and antigenic targets in ovarian pathology. Hum Reprod Update. 2004;10(2):163‐175. 15073145 10.1093/humupd/dmh014

[30] Edassery SL, Shatavi SV, Kunkel JP, et al Autoantigens in ovarian autoimmunity associated with unexplained infertility and premature ovarian failure. Fertil Steril. 2010;94(7):2636‐2641. 20522323 10.1016/j.fertnstert.2010.04.012 PMC2948062

[31] Meyers KE, Pfieffer S, Lu T, Kaplan BS. Genitourinary complications of systemic lupus erythematosus. Pediatr Nephrol. 2000;14(5):416‐421. 10805472 10.1007/s004670050786

[32] Wang X, Mao R, Wang M, Zhu L, Jin L. The genetic relationship between systemic lupus erythematosus and risk of primary ovarian failure from a Mendelian randomization study. Sci Rep. 2024;14(1):9413. 38658584 10.1038/s41598-024-59726-9 PMC11043424

[33] Hedrich CM . Shaping the spectrum—from autoinflammation to autoimmunity. Clin Immunol. 2016;165:21‐28. 26948930 10.1016/j.clim.2016.03.002

[34] Eaton WW, Rose NR, Kalaydjian A, Pedersen MG, Mortensen PB. Epidemiology of autoimmune diseases in Denmark. J Autoimmun. 2007;29(1):1‐9. 17582741 10.1016/j.jaut.2007.05.002 PMC2717015

[35] Miller FW . The increasing prevalence of autoimmunity and autoimmune diseases: an urgent call to action for improved understanding, diagnosis, treatment, and prevention. Curr Opin Immunol. 2023;80:102266. 36446151 10.1016/j.coi.2022.102266 PMC9918670

[36] Hemminki K, Li X, Sundquist K, Sundquist J. Shared familial aggregation of susceptibility to autoimmune diseases. Arthritis Rheum. 2009;60(9):2845‐2847. 19714648 10.1002/art.24749

[37] Cannon-Albright LA, Stevens J, Facelli JC, Teerlink CC, Allen-Brady K, Agarwal N. High-Risk pedigree study identifies LRBA (rs62346982) as a likely predisposition variant for prostate cancer. Cancers (Basel). 2023;15(7):2085. 37046747 10.3390/cancers15072085 PMC10092952

